# Abnormal Functional Connectivity of the Amygdala in Mild Cognitive Impairment Patients With Depression Symptoms Revealed by Resting-State fMRI

**DOI:** 10.3389/fpsyt.2021.533428

**Published:** 2021-07-15

**Authors:** Ting Yang, Bangli Shen, Aiqin Wu, Xinglu Tang, Wei Chen, Zhenzhong Zhang, Bo Chen, Zhongwei Guo, Xiaozheng Liu

**Affiliations:** ^1^The Second Affiliated Hospital and Yuying Children's Hospital, Wenzhou Medical University, Wenzhou, China; ^2^Department of Psychiatry, Sir Run Run Shaw Hospital, Zhejiang University School of Medicine, Hangzhou, China; ^3^Tongde Hospital of Zhejiang, Hangzhou, China

**Keywords:** mild cognitive impairment, depression, functional magnetic resonance imaging, functional connectivity, amygdala

## Abstract

Convergent evidence indicates that individuals with symptoms of depression exhibit altered functional connectivity (FC) of the amygdala, which is a key brain region in processing emotions. At present, the characteristics of amygdala functional circuits in patients with mild cognitive impairment (MCI) with and without depression are not clear. The current study examined the features of amygdala FC in patients with MCI with depression symptoms (D-MCI) using resting-state functional magnetic resonance imaging. We acquired resting-state functional magnetic resonance imaging data from 16 patients with D-MCI, 18 patients with MCI with no depression (nD-MCI), and 20 healthy controls (HCs) using a 3T scanner and compared the strength of amygdala FC between the three groups. Patients with D-MCI exhibited significant FC differences in the amygdala–medial prefrontal cortex and amygdala–sensorimotor networks. These results suggest that the dysfunction of the amygdala–medial prefrontal cortex network and the amygdala–sensorimotor network might be involved in the neural mechanism underlying depression in MCI.

## Introduction

Depression is a mood disorder characterized by negative emotional states and often coexists with mild cognitive impairment (MCI). The prevalence of depression in patients with MCI has been reported to range between 16.9 and 55%, which is higher than the range of 11–30% in normal adults (1, 2). Patients with MCI and depression have more cognitive deficits, such as lower processing speeds, worse executive functioning, and lower acquisition and retrieval of new information, than patients with MCI without depression (3, 4). Patients with MCI and depression have a significantly higher rate of annual conversion to Alzheimer's disease (AD) (31%) compared with patients with MCI without depression (13.5%) and the general population (4.2%) (3, 5). Patients with MCI and depression have more amyloid abnormalities than non-depressed patients, and the Aβ burden of the brain is associated with an increased risk of experiencing neuropsychiatric symptoms, including depression and progression to more severe cognitive impairment (5, 6). Additionally, the new onset of depression in patients with MCI has been associated with deep subcortical cerebral white matter hyperintensity (7). Therefore, depression in MCI is rooted in abnormal brain pathology, and it is important to explore the neural mechanism underlying depression in MCI (D-MCI) to facilitate the development of effective, targeted interventions.

The amygdala receives sensory information from the cortex, thalamus, hypothalamus, and brainstem (8). Previous studies have shown that amygdala dysfunction may be related to the pathogenesis of depression (9). Tang et al. (10) have also reported that the volume change rates of the left amygdala in patients with MCI and HCs were −1.32 ± 1.1% per year and −0.81 ± 0.73% per year, respectively. Furthermore, the volume change rates of the bilateral hippocampus and amygdala were negatively correlated with the Alzheimer's Disease Assessment Scale–Cognitive section (ADAS-cog) change rate. Therefore, abnormality of the amygdala functional network may underlie the extensive autonomic, emotional, and cognitive symptoms of depression. The medial prefrontal cortex (mPFC) also mediates emotional processing and is believed to be involved in cognition, attention regulation, and behavioral decision-making. mPFC dysfunction has been associated with an increase in negative self-processing bias in patients with depression (11, 12), and the CBF in the mPFC of patients with MCI was lower than in NCs (13). Xie et al. (14) found that the interactions between depressive symptoms and episodic memory deficits were associated with PFC volume loss.

Alterations of the amygdala–mPFC circuit have been widely reported in patients with depression (15–21). Using data from 100 fMRI experiments, one meta-analysis showed that hyperactivation of the mPFC is associated with diminished right amygdala activity during the regulation of negative emotions (12). Depressed adolescents have also been reported to exhibit reduced amygdala–mPFC FC during the emotional reappraisal of negative images (17). Similarly, depressed adults have been found to exhibit increased mPFC activity when observing happy faces and decreased mPFC activity when looking at sad faces, whereas this pattern was reversed in healthy adults (18). Previous work has also reported that decreased FC between the anterior cingulate cortex (ACC) and amygdala was associated with negative stimuli in unmedicated individuals with major depressive disorders (MDD) (19). Almeida and colleagues reported reduced amygdala–orbitofrontal cortex (OFC) FC during the processing of happy and sad faces in medicated patients with MDD (20). Using fMRI and diffusion tensor imaging data from 55 older adults with amnestic MCI, another study found that both functional and structural connectivities between the medial prefrontal cortex and amygdala were significantly correlated with external LOC (21). Using the coherence regional homogeneity method, we previously reported that D-MCI patients had abnormal temporal homogeneity compared with patients with no depression with MCI (nD-MCI) in the mPFC, post-central gyrus, and thalamus (22).

The aim of the present study was to examine alterations in amygdala FC in D-MCI patients using rsfMRI and seed-point FC by comparing with nD-MCI patients and healthy controls (HCs). Based on previous findings of abnormal amygdala FC in those with depression (15–21), we hypothesized that, relative to the nD-MCI and HC groups, the D-MCI group would display altered amygdala FC in regions of the mPFC that are critical to emotion regulation (23).

## Materials and Methods

### Patients

From July 2014 to August 2017, a total of 54 subjects were recruited (nD-MCI: *n* = 18; D-MCI: *n* = 16; HCs: *n* = 20) from the Tongde Hospital of Zhejiang Province, Hangzhou, Zhejiang, China. All subjects completed a battery of neuropsychological tests and clinical assessments. Written informed consent was obtained from all patients, and the study was approved by the Ethics Committee of Tongde Hospital of Zhejiang Province (approval no. 2017-11-12).

All the included subjects with MCI were right-handed, had a clinical dementia rating (CDR) of 0.5, and had a Mini-Mental State Examination (MMSE) score >24 (24). For HCs, the CDR was 0. Depressive symptoms were identified by professional psychiatrists according to the Diagnostic and Statistical Manual of Mental Disorders, fourth edition (25). The severity of depression was rated using the 17-item Hamilton Rating Scale for Depression (HAMD-_17_) (26) and the Neuropsychiatric Inventory (NPI) (27). Depression symptoms were considered present when the HAMD score was ≥7, and the NPI depression domain score was ≥4 (22, 28).

The exclusion criteria were as follows: left-handedness; lifetime history of psychiatric disorders, such as psychotic or bipolar disorders; taking antidepressant drugs; and MR imaging contraindications.

### MRI Scanning

The MRI scan was performed using a 3.0-Tesla Siemens scanner (Siemens Magnetom Verio, Siemens Medical Systems, Erlangen, Germany). Participants were instructed to rest with their eyes closed during resting-state scans. rsfMRI was performed using an echo-planar imaging sequence, as follows: 33 axial slices, thickness/gap = 4.8/0 mm, in-plane resolution = 64 × 64, repetition time (TR) = 2,000 ms, echo time (TE) = 30 ms, flip angle = 90°, and field of view (FOV) = 200 × 200 mm^2^, 200 volumes. High-resolution T1-weighted images were acquired using a 3D MPRAGE sequence with the following parameters: TI/TR/TE = 900/1,900/2.48 ms, flip angle = 9°, 128 slices, FOV = 256 × 256 mm^2^, 1 × 1 × 1 mm^3^ resolution for each subject.

### Data Processing

Data preprocessing was conducted using the DPABI toolbox v4.0 (29). Preprocessing steps involved deleting the first 10 volumes, slice timing, and motion correction. Participants with a low framewise displacement of head motion (mean FD <0.5) were included (30). To normalize fMRI images to the Montreal Neurological Institute (MNI) space, we first co-registered fMRI images to each individual's high-resolution T1 anatomical scan and further normalized them to the MNI152 template. Then, the normalized images were smoothed with a 6-mm full-width at half-maximum Gaussian kernel, linear detrending, and temporal bandpass filtering (0.01–0.08 Hz). To remove spurious signals, head motion artifacts, cerebrospinal fluid (CSF), and white matter were regressed out of each voxel's time series. We did not remove the global signal because this is still a controversial issue (31).

### Amygdala Resting-State Functional Connectivity Analysis

For each participant, two separate amygdala seeds (one per hemisphere) were created on the AAL template using an atlas-based method (32). The Pearson's correlation coefficients between the average seed time series and the time series of whole-brain voxels were computed. Fisher's *Z*-transform was performed on Pearson's correlation maps to verify the normal distribution of correlation images.

### Statistical Analysis

Statistical analyses of the demographics and clinical characteristics were performed using the Statistical Package for the Social Sciences v.15.0 software (SPSS Inc., Chicago, IL, USA). Two-sample *t*-tests were carried out to determine between-group differences in age and education, and chi-square tests were applied to determine between-group differences in sex distribution. We analyzed the left and right amygdala FC maps separately using DPABI. A one-way ANCOVA was conducted to compare the individual normalized resting-state functional connectivity (RSFC) maps in a voxel-by-voxel manner between the three groups. Then, we extracted brain masks that showed significant differences in the ANCOVA, corresponding to the left and right amygdala FC maps, separately for each hemisphere. Finally, we carried out *post-hoc t*-tests between each pair of groups based on the ANCOVA brain masks. To ensure the certainty of the results, we regressed out the mean relative displacements of head motion, age, and sex as covariates in the ANCOVA and two-sample *t*-tests. For multiple comparisons, we calculated the FWHM of the smoothing kernel on residual images using 3dFWHMx; the estimated FWHM value was 10.96 mm. The significance level was set at *p* < 0.05 (3dClustSim-corrected for multiple comparisons, with an individual voxel *p* < 0.05 with a cluster size >418 voxels).

### Relationship of FC With Clinical Variables

To examine the association of neuropsychological performance with FC values in D-MCI and nD-MCI patients, we extracted the mean z-values of the abnormal brain regions and conducted Pearson's correlation analyses (*p* < 0.05).

### Feature Selection and Model Validation

To evaluate the amygdala FC as feature sets for distinguishing D-MCI from nD-MCI, we constructed a support vector machine (SVM) classification experiment using MATLAB R2014a. We extracted the mean FC signals of different brain regions in D-MCI and nD-MCI groups (**Table 2**). These mean FC signals were used as features for SVM training and classification. We constructed an SVM model using the MATLAB script fitcsvm. Given the small sample size, we validated the classifier using 10-fold cross-validation using the MATLAB script crossval. The training parameters of the SVM model were the default parameters provided by MATLAB 2014a. We also computed the following corresponding evaluation indices: accuracy (ACC), sensitivity (SEN), specificity (SPE), area under the receiver operating characteristic curve (AUC), and F-score (33).

## Results

### Neuropsychological Results

The demographics and clinical data are shown in Table 1. There were no significant between-group differences in age (ANOVA: *F* = 0.287, *p* = 0.510), sex distribution (chi-square test: c2 = 6, *p* = 0.199), or education level (ANOVA: *F* = 0.266, *p* = 0.726). The D-MCI group had significantly higher D-NPI and HAMD scores than the nD-MCI group (ANOVA: *F* = 154.84, *p* < 0.001; *F* = 203.07, *p* < 0.001). There were no significant differences in the MMSE score between the nD-MCI and D-MCI groups (*t*-tests: *T* = 0.037, *p* = 0.971). There were no significant differences in the HAMD score (*t*-tests: *T* = 1.035, *p* = 0.3430) or the NPI score (*t*-tests: *T* = 1.303, *p* = 0.1952) between the nD-MCI and HC groups.

**Table 1:** Demographics and neuropsychological data.

	**D-MCI group**	**nD-MCI group**	**HC group**	***F/c2* value**	***P-*value**
Gender, *n* (M/F)	16 (6/10)	18 (7/11)	20(11/9)	6	0.199
Age, years	69.6 ± 6.2	72.1 ± 9.7	72.3 ± 6.08	0.287	0.510
Education, years	8.3 ± 2.1	8.5 ± 1.8	7.80 ± 2.04	0.266	0.726
MMSE	26.6 ± 1.1	26.6 ± 1.0	29.1 ± 0.90	36.89	<0.001
HAMD	11.7 ± 3.1	2.00 ± 1.73	1.50 ± 1.43	203.07	<0.001
D-NPI	7.19 ± 2.3	0.33 ± 0.48	0.15 ± 0.36	154.84	<0.001

*Data are represented as the mean ± SD. The chi-square test was used to compare sex, and a one-way ANOVA with post-hoc two-sample t-tests was used to compare age and neuropsychological data. HC, healthy controls; D-MCI, mild cognitive impairment with depression; nD-MCI, no depression and mild cognitive impairment; M, male; F, female; MMSE, Mini-Mental State Examination; D-NPI, depression domain of the Neuropsychiatric Inventory; HAMD, Hamilton Depression Rating Scale*.

### Abnormal Amygdala FC Values in the D-MCI Group

**Figure 1:**
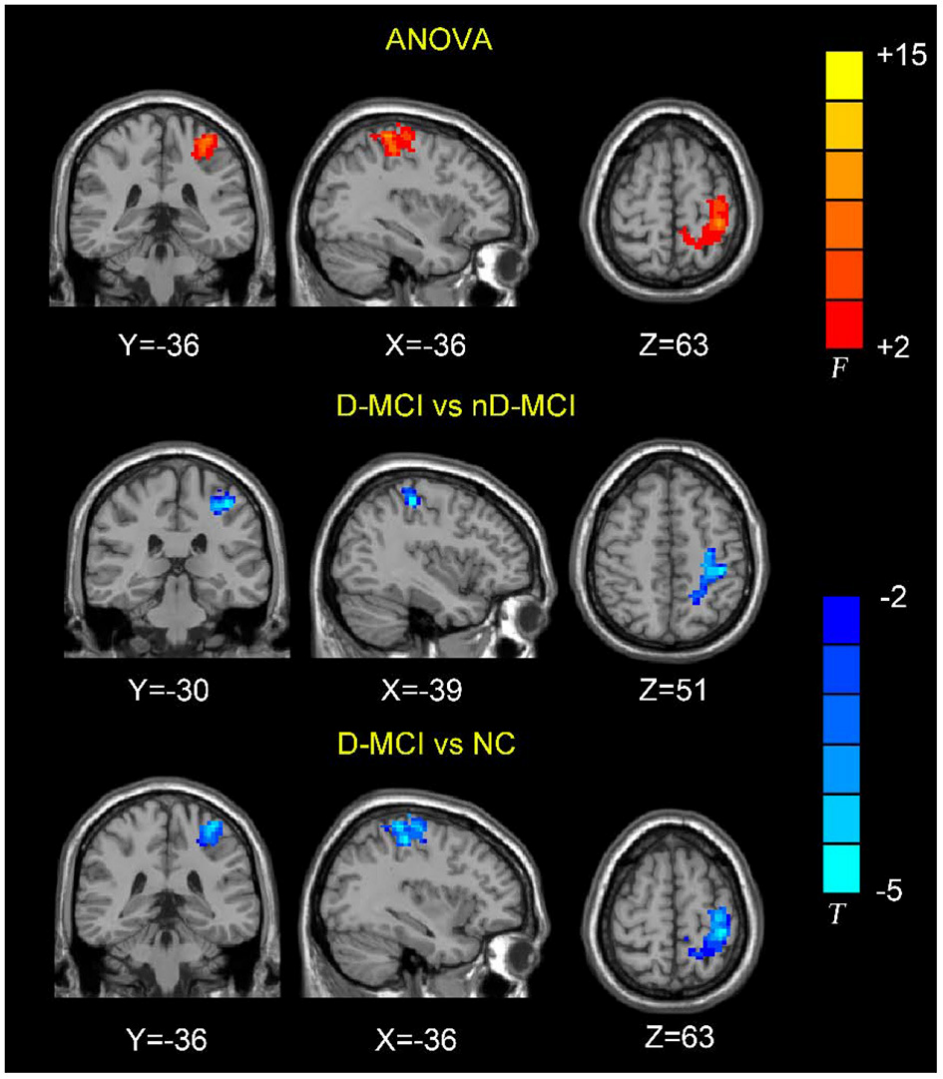
Brain regions showing the decreased left amygdala functional connectivity (FC) values in the D-MCI compared with nD-MCI and HC groups.

**Figure 2:**
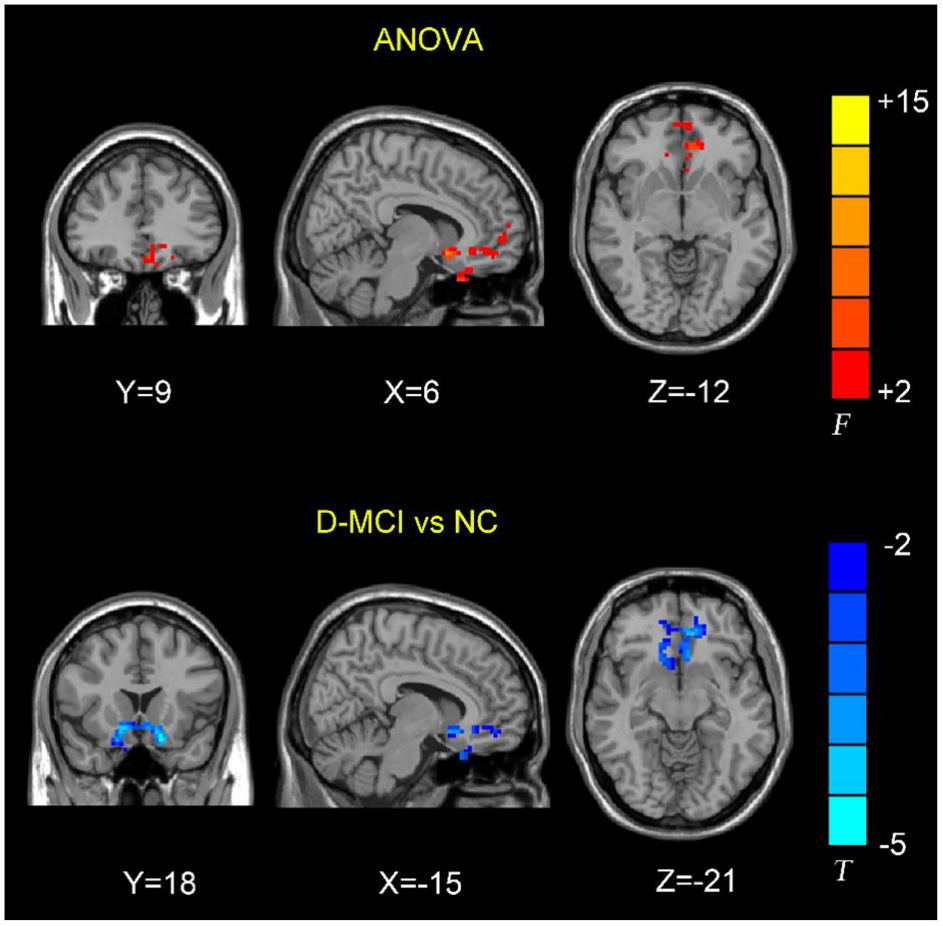
Brain regions showing the decreased right amygdala functional connectivity (FC) values in the D-MCI compared with nD-MCI and HC groups.

The between-group analysis revealed bilateral amygdala connectivity in the D-MCI group relative to the nD-MCI and HC groups across multiple regions, except for left amygdala connectivity with the left post-central gyrus (Figure 1 and Table 2) and right amygdala connectivity with the left ACC, right OFC, and mPFC (Figure 2 and Table 3).

**Table 2:** Brain regions with significantly different FC values with the left amygdala in the D-MCI group compared with the nD-MCI group and HC group.

**Brain regions**	**Voxels**	**BA**	**MNI coordinates**			***F/T* value**	***P*-value**
			***x***	***y***	***z***		
**ANOVA**							
Post-central_L	498	3	−36	−36	63	9.6240	0.0466
**D-MCI vs. nD-MCI**							
Post-central_L	513	3	−39	−30	51	−3.5103	0.0362
**D-MCI vs. NC**							
Post-central_L	911	3	−36	−36	63	−4.1349	0.0269

*D-MCI, mild cognitive impairment with depression; nD-MCI, no depression with mild cognitive impairment; MNI, Montreal Neurological Institute; BA, Brodmann area*.

**Table 3:** Brain regions with significantly different FC values with the right amygdala in the D-MCI group compared with the nD-MCI group and HC group.

**Brain regions**	**Voxels**	**BA**	**MNI coordinates**			***F/T*-value**	***P-*value**
			***x***	***y***	***z***		
**ANOVA**							
Olfactory_R	473	3	6	9	−12	8.7981	0.0487
Cingulun_Ant_L		11	−8	40	−4	3.4630	0.3710
Frontal_Sup_Medial_L		10	−13	55	4	3.4533	0.3730
**D-MCI vs. NC**							
Frontal_Sup_Orb_R	612	11	−15	18	−21	−4.2927	0.0251
Cingulum_Ant_L		10	−12	41	−5	−2.7001	

*D-MCI, mild cognitive impairment with depression; nD-MCI, no depression with mild cognitive impairment; MNI, Montreal Neurological Institute; BA, Brodmann area*.

### Correlations of Amygdala FC With Clinical Variables

We found no significant correlations between the amygdala FC values and the D-NPI or HAMD scores.

### Classification Performance of Amygdala FC

**Figure 3:**
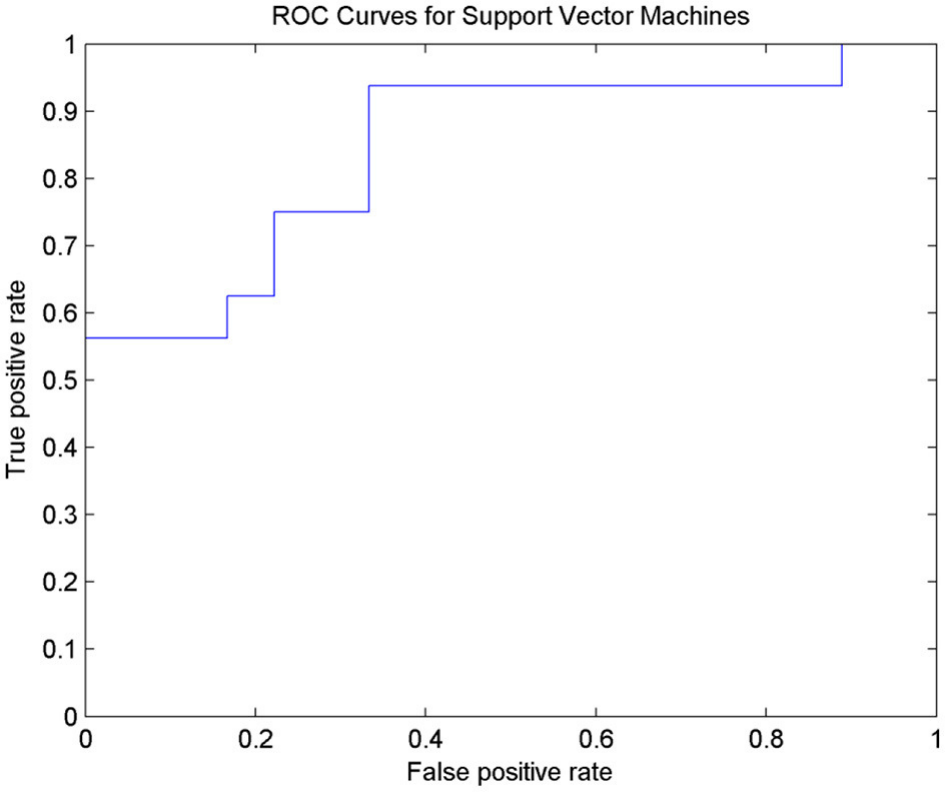
ROC curves for SVM classification to distinguish D-MCI and nD-MCI patients.

After 10-fold cross-validation, the SVM model showed high diagnostic performance with an AUC value of 0.8438. The SVM model had relatively good accuracy in discriminating nD-MCI and relatively weak accuracy in discriminating D-MCI (Figure 3 and Table 4).

**Table 4:** Predictive performance table for SVM.

	**AUC**	**ACC**	**SEN**	**SPE**	**F-score**
SVM	0.8438	0.7647	1	0.6923	0.6667

*ACC, accuracy; SEN, sensitivity; SPE, specificity; AUC, area under the receiver operating characteristic curve*.

## Discussion

In this study, we examined resting-state amygdala FC in D-MCI, nD-MCI, and HC groups using rsfMRI data. Compared with the nD-MCI and HC groups, the D-MCI group showed reduced left amygdala FC with the left post-central gyrus. Compared with the HC group, the D-MCI group had reduced right amygdala FC with the left ACC, right OFC, and mPFC.

We found an altered FC of the right amygdala with the pregenual ACC and its adjacent brain regions, including the OFC and mPFC, which are involved in the affective network (AN) and cognitive control network (CCN) (23, 34). Previous studies have demonstrated that depressed adolescents exhibit decreased right amygdala resting-state functional connectivity with frontal cortical areas, including the ACC (35), and lower fractional anisotropy in the white matter tract connecting the ACC to the amygdala in the right hemisphere (36). Using task-based fMRI data, Perlman et al. also reported that adolescents with MDD exhibit less amygdala–mPFC connectivity during emotion regulation (17). Wang et al. further investigated the FC patterns and altered functional interactions between the AN and CCN in patients with MDD (37). The authors found that, compared with HCs, patients with MDD showed reduced FC between the amygdala and the right ACC within the AN and reduced FC between the right DLPFC and the right ACC within the CCN. The ACC is the interaction hub of altered FC in MDD between the AN and the CCN. Further correlation analysis showed that the altered FC between the right ACC and amygdala was negatively correlated with the depressive symptom score, while the altered FC between the right ACC and DLPFC was positively correlated with the executive function in patients with MDD. Thus, our findings of the decreased amygdala–ACC connectivity in patients with D-MCI suggest that poor top-down regulation of the amygdala by the ACC may hinder effective emotion regulation.

We also found altered FC between the amygdala and the post-central gyrus, which is part of the sensorimotor network (SMN). The somatosensory-related cortices are considered to not only underlie the sensation of the body but also extract the social information that is required to understand emotions (38). Using data from a large rsfMRI dataset of the Human Connectome Project (820 participants), Toschi et al. (39) found the existence of a distinct amygdala–sensory/(pre)motor functional network during rest. Also, an fMRI attentional task that incorporated emotional pictures revealed that patients with MDD exhibited increased activation in the post-central gyrus in response to sad pictures (40). After escitalopram oxalate treatment, brain activity in the bilateral post-central gyrus was increased during emotion recognition in first-episode treatment-naïve patients with MDD (41). Studies have also found that individuals with PTSD show greater amygdala–somatosensory cortex FC when exposed to trauma-related stimuli and when remembering negative emotional autobiographical memories compared with healthy trauma-exposed and non-trauma-exposed adults (42, 43). More negative amygdala–post-central gyrus FC during rest, as well as during recall of the trauma memory, predicted the 6-month incidence of PTSD (44). These findings suggest that dysfunction of the amygdala–SMN network is related to emotion dysregulation in patients with D-MCI.

The alterations in FC of the left amygdala with the post-central gyrus and right amygdala with the OFC indicate that there are D-MCI-induced asymmetric changes in the amygdala–cerebral cortex networks, as shown by previous studies (45, 46). Zotev et al. (45) reported that patients with MDD had asymmetric EEG changes in the frontal cortex during online fMRI neurofeedback tasks, which were positively correlated with depression severity. Bartholomeusz et al. (46) investigated the relationship between amygdala volume asymmetry and emotion recognition impairments in ultra-high risk for psychosis (UHR) individuals. The authors found that the amygdala volume was positively associated with sadness emotion recognition and that the left amygdala volume mediated the influence of sad mood recognition on depressive symptoms. Furthermore, a low persistence (PS) affective style has been associated with a greater vulnerability to depression; Sterpenich et al. (30) found that low PS individuals exhibited higher amygdala and right OFC activity, but lower left OFC activity, when processing negative pictures, compared with high PS individuals. This prefrontal cortex asymmetry indicates that low PS individuals have a stronger avoidance response to aversive stimuli. Our results suggest that this asymmetry may represent a neural mechanism underlying aberrant cognitive and emotional regulation in patients with D-MCI.

We did not perform scrubbing during the fMRI preprocessing because doing so is still a controversial issue. Scrubbing is a method to remove epochs in which large head movements are detected (47, 48) and has shown some effectiveness in removing spurious sources of connectivity in fMRI data. However, it can also have unintended consequences. For example, filters that are applied after scrubbing will not correctly work because filters have a temporal dependency. Moreover, a larger number of scrubbing spikes in the fMRI data will systematically reduce time autocorrelations (49).

We found no correlation between the amygdala-FC and the neuropsychological performance of cognition and emotion. Previous studies have shown similar results (50–60). Zheng et al. (50) found that MCI with depression displayed increased FC from the amygdala to the lingual, calcarine gyrus, and supplementary motor areas, compared with MCI and HC; however, they did not find a correlation between amygdala connectivity and the scores of the mini-mental state examination. Yuan et al. (51) also failed to find a significant association between Montreal Cognitive Assessment scores and the amplitude of low-frequency fluctuation signal maps after 4 weeks of repetitive transcranial magnetic stimulation on MCI. A few studies of identifying the brain FC alterations during different stages of AD suggest that dysfunction of more resting-state networks accompanies the progression of AD (52), and AD is associated with widespread loss of both intranetwork and internetwork correlations (53, 54). Ortner et al. studied the progressively disrupted intrinsic FC of the amygdala in very early AD. Their results showed that MCI reduced the positive amygdala FC with parieto-occipital regions, insula, and hippocampus; mild AD patients reduced the amygdala FC mainly in the medial temporal and insular regions (55). These studies show that the severity of symptoms is the result of multiple neural network changes, not necessarily parallel to a single network change (56–58). This may be one of the reasons why we did not find a correlation between the amygdala-FC and neuropsychological performance. Another reason may be our choice of technical parameters, frequency bands (0.01–0.08 Hz). Yang et al. (59) computed FC patterns across slow-5 (0.01–0.027 Hz) and slow-4 (0.027–0.073 Hz) bands in bipolar disorder during depressive episodes (BDD). In the slow-4 band, the BDD patients showed increased FC in the left fusiform gyrus (FG) and the left lingual gyrus (LG). In the slow-5 band, the BDD patients showed decreased FC in the left LG. The increased FC in the left FG in the slow-4 band was correlated with clinical progression. Qin et al. (60) also explored the topological properties of AD patients in the specific frequency band; they found that the global efficiency, the “small-world” properties of AD patients, decreased at lower-frequency bands (0.01–0.06 and 0.06–0.11 Hz). However, at higher-frequency bands (0.11–0.25 Hz), the characteristic path length was much longer, and the “small-world” property was disrupted in AD. Their results suggested that the topological alterations of large-scale functional brain networks in AD patients are frequency dependent.

There are some limitations to our study that should be noted. First, the patient sample size was small, which may have affected the statistical power and increased the possibility of false-positive results. Second, different subregions of the amygdala underlie different functions. Further studies should therefore investigate FC in different subregions of the amygdala (61). Third, this was a cross-sectional study, and long-term follow-up studies could be performed in the future to test the correlation between the amygdala-FC and the reduction of depression symptoms. Finally, we did not perform a clinical alcohol test when we recruited the subjects; recent studies have shown that alcohol abuse is associated with dysfunction of amygdala networks (62). Future studies could therefore collect data on alcohol intake and include this variable as a covariate in the data analysis.

## Conclusions

In the current study, we compared the FC of the amygdala in D-MCI, nD-MCI, and HC groups. Our results showed that patients with D-MCI exhibited significant RSFC differences in the amygdala–mPFC network and the amygdala–sensorimotor network. These findings enhance our understanding of the functional mechanism of the amygdala network in patients with D-MCI.

## Data Availability Statement

The datasets generated for this study are available on request to the corresponding author.

## Ethics Statement

The studies involving human participants were reviewed and approved by Tongde Hospital of Zhejiang Province. The patients/participants provided their written informed consent to participate in this study.

## Author Contributions

AQW, ZWG, and WC designed the study. ZZZ, XLT, and AQW performed the experiments. XZL and TY analyzed the data. XZL, ZWG, BLS, and BC drafted the manuscript. TY, ZZZ, XZL, ZWG, and BC revised the manuscript and final approved of the version to be published. All authors participated in the discussion and writing of the manuscript.

## Conflict of Interest

The authors declare that the research was conducted in the absence of any commercial or financial relationships that could be construed as a potential conflict of interest.
